# Identification of cancer hallmark‐associated gene and lncRNA cooperative regulation pairs and dictate lncRNA roles in oral squamous cell carcinoma

**DOI:** 10.1111/jcmm.15174

**Published:** 2020-03-23

**Authors:** Ken Lin, Lin‐Jing Song, Jing Ma, Tie‐Song Zhang, Ding‐Yun You, Yong‐Wen He

**Affiliations:** ^1^ Department of Otolaryngology, Head and Neck Surgery The Affiliated Children's Hospital of Kunming Medical University Kunming China; ^2^ Department of Otolaryngology, Head and Neck Surgery Kunming Children's Hospital Kunming China; ^3^ The Affiliated Stomatological Hospital of Kunming Medical University Kunming China; ^4^ Department of Oncology Yan'an Hospital Kunming China; ^5^ School of Public Health Kunming Medical University Kunming China; ^6^ Department of Dental Research The Affiliated Stomatological Hospital of Kunming Medical University Kunming China

**Keywords:** cancer hallmark, co‐mutation, cooperative regulation pairs, lncRNA, oral squamous cell carcinoma, prognosis

## Abstract

Oral squamous cell carcinoma (OSCC) is the most common malignant tumour in the oral and maxillofacial region. Numerous cancers share ten common traits (“hallmarks”) that govern the transformation of normal cells into cancer cells. Long non‐coding RNAs (lncRNAs) are important factors that contribute to tumorigenesis. However, very little is known about the cooperative relationships between lncRNAs and cancer hallmark‐associated genes in OSCC. Through integrative analysis of cancer hallmarks, somatic mutations, copy number variants (CNVs) and expression, some OSCC‐specific cancer hallmark‐associated genes and lncRNAs are identified. A computational framework to identify gene and lncRNA cooperative regulation pairs (GLCRPs) associated with different cancer hallmarks is developed based on the co‐expression and co‐occurrence of mutations. The distinct and common features of ten cancer hallmarks based on GLCRPs are characterized in OSCC. Cancer hallmark insensitivity to antigrowth signals and self‐sufficiency in growth signals are shared by most GLCRPs in OSCC. Some key GLCRPs participate in many cancer hallmarks in OSCC. Cancer hallmark‐associated GLCRP networks have complex patterns and specific functions in OSCC. Specially, some key GLCRPs are associated with the prognosis of OSCC patients. In summary, we generate a comprehensive landscape of cancer hallmark‐associated GLCRPs that can act as a starting point for future functional explorations, the identification of biomarkers and lncRNA‐based targeted therapy in OSCC.

## 1 INTRODUCTION

Oral squamous cell carcinoma (OSCC) is the most common malignant tumour in the oral and maxillofacial region, with 4 260 000 new cases arising and ~128 000 deaths occurring annually.^1^, ^2^ The incidence of OSCC has increased in many countries and especially in young people.^3^ Many kinds of pathogenic factors, including smoking, alcohol abuse and human papillomavirus infection, all accelerate the tumorigenesis of OSCC.^4^ Five‐year survival rates for OSCC patients are greater than 80%; however, they drop dramatically to 40% for patients with lymph node involvement and 20% for patients with distant metastasis.^5^, ^6^, ^7^ The standard treatment for OSCC is surgery, often accompanied by radiotherapy and chemotherapy.^8^ There are no effective and reliable biomarkers that predict the aggressiveness or treatment response of OSCC patients.

Long non‐coding RNAs (lncRNAs) are a type of non‐coding RNA with 200 nucleotides and do not code for proteins.^9^ lncRNAs are pervasive across the genome, and dysregulation of their expression is associated with various human diseases including cancer.^10^, ^11^, ^12^ For example, the lncRNA CTS promotes metastasis in cervical cancer.^13^ The lncRNA MYOSLID functions as a competing endogenous RNA to regulate MCL expression by sponging miR‐29c‐3p in gastric cancer.^14^ Some studies have shown that lncRNAs play vital roles in OSCC. For example, the lncRNA SNHG20 promotes the tumorigenesis of OSCC via targeting the miR‐197/LIN28 axis.^15^ The lncRNA p23154 promotes the invasion‐metastasis potential of OSCC by regulating Glut1‐mediated glycolysis.^16^ The lncRNA PDIA3P interacts with miR‐185‐5p to modulate OSCC progression by targeting Cyclin D2.^17^ Moreover, lncRNA and genes can cooperate to fulfil their functions in diseases.^18^, ^19^, ^20^ Studying the cooperation between genes and lncRNA is an effective way to predict the roles of lncRNA in disease according to the function of their cooperated genes. For example, lncRNA ANRIL supports proliferation of adult T‐Cell leukaemia cells through cooperation with gene EZH2.^19^ Zhu et al^21^ analysed a lncRNA and gene co‐expression network to identify cycle‐related lncRNAs in hepatocellular carcinoma. These kinds of regulatory pairs are named gene and lncRNA cooperative regulation pairs (GLCRPs). However, most studies are only focused on co‐expression.

A hallmark is usually defined as “a feature of something that distinguishes it from others.”^22^ In 2000, Hanahan and Weinberg proposed six hallmarks of cancer, and in 2011, they extended the cancer hallmarks to a list of ten.^23^, ^24^ They suggest that all kinds of cancer share ten common traits (“hallmarks”) that govern the transformation of normal cells to cancer cells. These hallmarks are ten underlying principles shared by all cancers, which provide a useful framework to understand the remarkable diversity of cancer. However, we have little insight into how to use these hallmarks to depict the roles of lncRNAs and provide biomarkers and treatment for OSCC.

In the present study, an integrated approach is developed to identify GLCRPs associated with cancer hallmarks in OSCC based on mutational and expression data. Some context‐specific genes and lncRNAs are discovered in OSCC. We systematically identify and analyse cancer hallmark‐associated GLCRPs in OSCC. The genes and lncRNAs in GLCRPs show similar mutation and expression patterns. The distinct and common features for ten cancer hallmarks based on GLCRPs are characterized in OSCC. Some key GLCRPs participate in many cancer hallmarks in OSCC. Cancer hallmark‐associated GLCRP networks show complex patterns and specific functions in OSCC. Specifically, some key GLCRPs are associated with the prognosis of OSCC patients. Overall, GLCRPs may accelerate biomarker discovery and therapeutic development in OSCC.

## 2 MATERIALS AND METHODS

### 2.1 GO terms and genes associated with cancer hallmarks

Cancer hallmark‐related GO terms were obtained from a previous study^25^ (Table S1). All the genes in these cancer hallmark‐related GO terms were downloaded from Gene Ontology using AmiGo.^26^ Thus, cancer hallmark‐related genes were obtained for subsequent analysis.

### 2.2 Mutation and expression profiles of genes and lncRNAs in OSCC

The major mutation data in the present study include somatic mutations and copy number variants (CNVs). We obtained CNV (level 3), somatic mutation (level 3), lncRNA expression and gene expression (level 3) data, as well as clinical data of OSCC patients, from The Cancer Genome Atlas (TCGA, Release: 2017‐09‐08). The genome annotation data, including genome sites and symbols of genes and lncRNAs, were downloaded from GENCODE 31 (19.06.19). Lastly, 329 tumour and 32 normal control OSCC samples were included in our analysis. The validated data set http://www.ncbi.nlm.nih.gov/geo/query/acc.cgi?acc=GSE30784 was downloaded from Gene Expression Omnibus (GEO). The data set included 167 OSCC and 45 normal oral tissues. To filter lncRNA and genes not expressed across all samples, the items with expression values of 0 in all of the samples were excluded. Any remaining expression values of 0 were set to the minimum value of all samples, and all values were log2‐transformed. All the download links were included in Table S2.

### 2.3 Identification of OSCC‐specific cancer hallmark‐related genes and lncRNAs

First, we identified OSCC‐specific cancer hallmark‐related genes. The genes and lncRNA expression profiles were generated by log transformation to construct expression profiles that followed normal distribution. *t* Tests were used to calculate the differential expression of all hallmark‐related genes between OSCC and normal control samples. The false discovery rate (FDR) was calculated for *P*‐values of the *t* tests. The somatic mutation and CNV levels were obtained for each cancer hallmark‐related gene in OSCC samples. A cancer hallmark‐related gene was considered an OSCC‐specific cancer hallmark‐related gene if it was differentially expressed (FDR < 0.01) and contained some mutations (CNV or more than three somatic mutations). The OSCC‐specific lncRNAs were obtained based on a similar pipeline.

### 2.4 Identification of GLCRPs in OSCC based on mutation and expression data

We considered that a gene and a lncRNA that cooperated to play their role, called a GLCRP, should show correlations at both the mutation and expression levels. First, Pearson's correlation coefficients (PCCs) were calculated for each OSCC‐specific gene and lncRNA pair. The gene and lncRNA were considered a co‐expressed pair if the PCC was higher than 0.3 or smaller than −0.3 and the *P*‐value was smaller than .01. Second, we tested the co‐occurrence of mutations for each candidate gene and lncRNA pair. Co‐occurrence was considered if there were concurrent mutations in the same samples for genes and lncRNAs. Fisher's exact test was used to identify co‐occurrence gene and lncRNA pairs based on two‐by‐two contingency tables. For each gene and lncRNA pair, we quantified the number of samples with (a) common mutations, (b) the gene mutation only, (c) the lncRNA mutation only and (d) mutations other than the pair examined. These four values were used to calculate the odds ratio using Fisher's exact test. The gene and lncRNA pairs were considered co‐occurrence pairs if the *P*‐values of Fisher's exact test were smaller than .01. Thus, GLCRPs for OSCC were identified based on the co‐expression and co‐occurrence of mutations.

GLCRP networks for each cancer hallmark in OSCC were constructed by Cytoscape 3.3.0 (http://www.cytoscape.org/). The degree analysis of each network was also performed using Cytoscape 3.3.0.

### 2.5 Survival analysis for the GLCRP in each cancer hallmark for OSCC

We used the regression coefficient of genes and lncRNAs in the cancer hallmark‐related GLCRPs related to patient survival based on gene and lncRNA expression data to verify if these cancer hallmark‐related GLCRPs were associated with survival. First, we randomly divided the OSCC patient samples into two independent groups. Second, a multivariate Cox regression model was performed for genes and lncRNA in each GLCRP to obtain a standardized Cox regression coefficient for the first group. Some confounders, including age, cancer stage and sex, were also considered in this process. A risk‐score formula was established for each OSCC patient based on the expression values of the genes and lncRNAs for the held‐out group weighed by their estimated regression coefficients, following the above multivariate Cox regression analysis. Performing Cox regression analysis and validating the model in two independent data sets are helpful for avoiding overfitting. Third, the OSCC patients were divided into high‐ and low‐risk groups based on the median of the risk score as the threshold value. Finally, we performed Kaplan‐Meier survival analysis for the high‐ and low‐risk groups. The statistical significance was assessed using the log‐rank test. Cancer hallmark‐related GLCRPs were significantly associated with survival when *P*‐value <.05. All analyses are performed within the R 3.3.3 framework.

## 3 RESULTS

### 3.1 Some context‐specific genes and lncRNAs based on cancer hallmarks were discovered in OSCC

**FIGURE 1:**
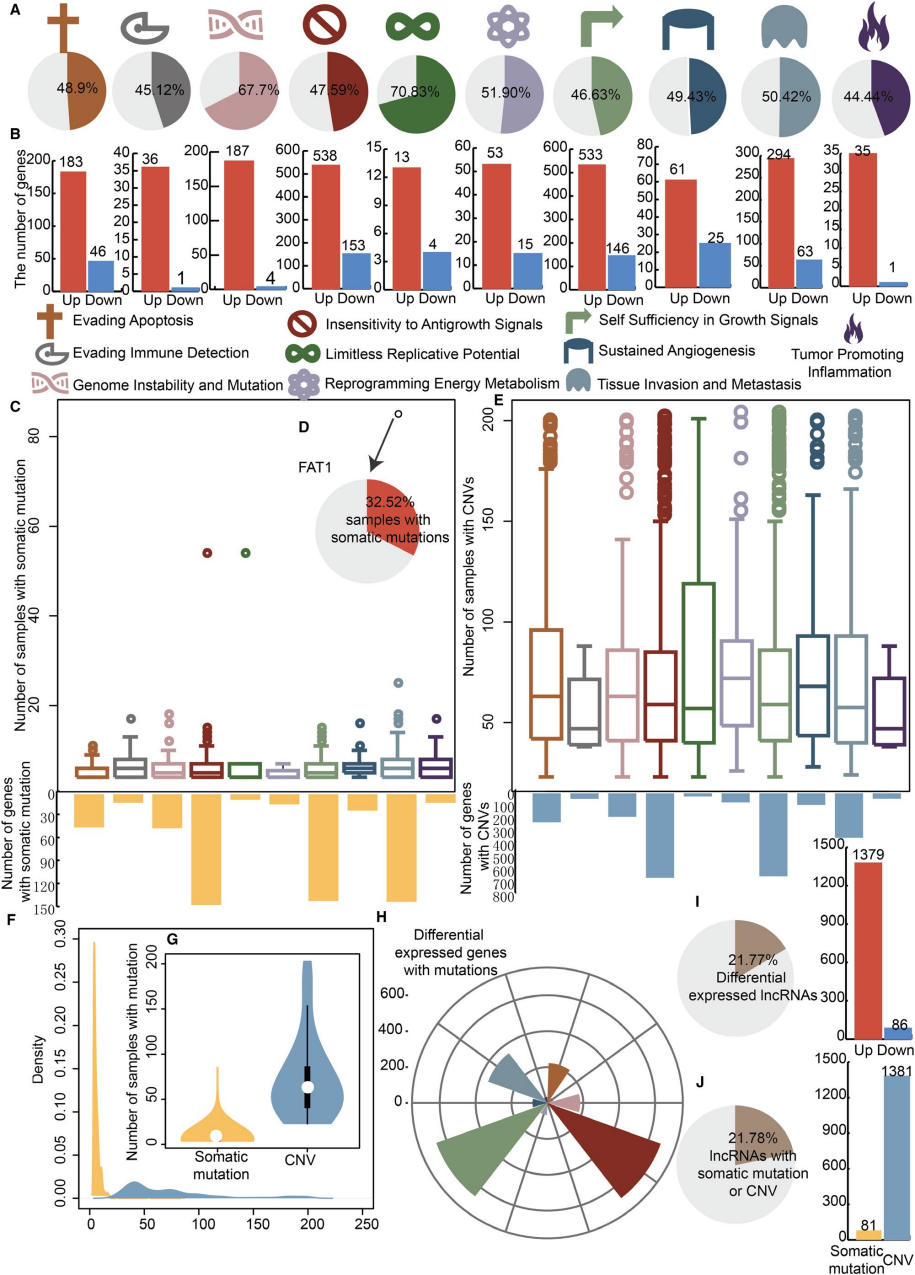
OSCC‐specific cancer hallmark‐associated genes and lncRNAs. A, The pie charts show the percentages of differentially expressed genes in each cancer hallmark. B, The bar plots show up‐ and down‐regulated differentially expressed genes in each cancer hallmark. C, The box plots show the distribution of the number of samples with somatic mutations in each cancer hallmark. The bar plots show the number of genes with somatic mutations in each cancer hallmark. D, The pie chart shows the percentages of samples with somatic mutations in the gene FAT1. E, The box plots show the distribution of the number of samples with CNVs in each cancer hallmark. The bar plots show the number of genes with CNVs in each cancer hallmark. F, The density distribution curves show the distribution of somatic mutations (yellow) and CNVs (blue) in OSCC. G, The violin plots show the number of samples with mutations. H, The rose diagram shows the numbers of differentially expressed genes with mutations in each cancer hallmark for OSCC. I, The pie chart shows the percentages of differentially expressed lncRNAs. The bar plot shows the up‐ and down‐regulated differentially expressed lncRNAs. J, The pie chart shows the percentages of lncRNAs with somatic mutations and CNVs. The bar plot shows the lncRNAs with somatic mutations and CNVs

To identify OSCC‐specific cancer hallmark‐related genes and lncRNAs, we considered both the expression and mutation levels of genes and lncRNAs in OSCC patients. For each cancer hallmark, there were 44.44%‐70.83% differentially expressed genes (Figure 1A). The cancer hallmark limitless replicative potential had the most differential genes in all cancer hallmarks (70.83%). The regulation directions, including up‐ and down‐regulated differential genes in cancer hallmarks, were diverse (Figure 1B). Most differential genes were up‐regulated in all kinds of cancer hallmarks for OSCC patients. We largely considered two kinds of mutation, point somatic mutations and CNVs, in OSCC. We considered a gene to be a mutated gene in OSCC if somatic mutations occurred in more than three samples or CNVs in more than one sample. The numbers of samples with somatic mutations were concentrated around ten for mutated genes in each cancer hallmark (Figure 1C). There were some mutated genes with high somatic mutation frequencies. For example, in the FAT1 gene, somatic mutation occurred in 32.52% of OSCC samples (Figure 1D). The mutation frequency of CNV was concentrated on 100 for each cancer hallmark in OSCC (Figure 1E). The density distributions of somatic mutations and CNVs were focused on diverse regions in OSCC (Figure 1F). The mutation frequency of CNV was significantly improved relative to the somatic mutations for each cancer hallmark in OSCC (Figure 1G). We extract the OSCC‐specific cancer hallmark‐related genes if the genes were both differentially expressed and mutated genes. There were 16‐661 OSCC‐specific genes in diverse cancer hallmarks (Figure 1H). OSCC‐specific lncRNAs were also identified. There were 21.77% differential lncRNAs, and most of them were up‐regulated in OSCC (Figure 1I). There were 21.78% lncRNAs with somatic mutations or CNV (Figure 1J). Similar to genes, the frequency of CNV in lncRNAs was higher than that of somatic mutations.

### 3.2 Systematic identification of cancer hallmark‐associated GLCRPs in OSCC

**FIGURE 2:**
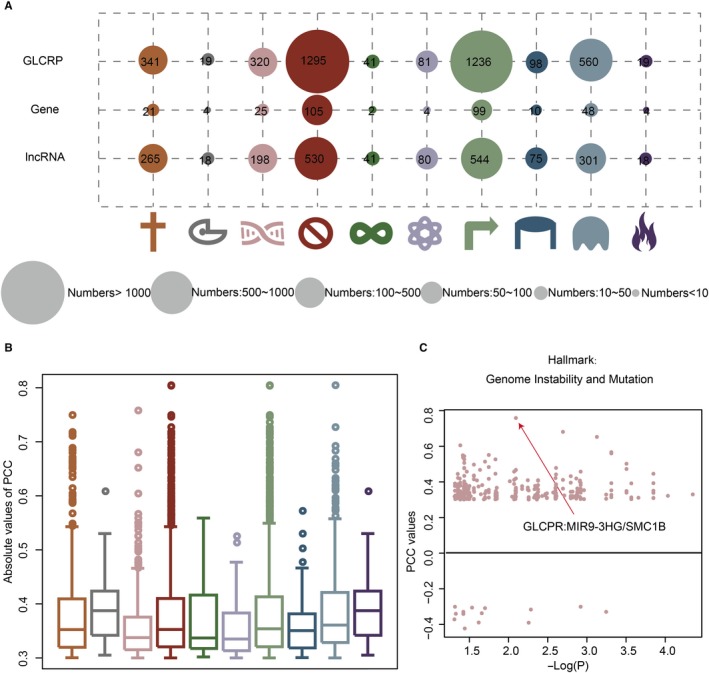
Cancer hallmark‐associated GLCRPs in OSCC. A, The size of the circle represents the number of GLCRPs, genes and lncRNAs in each cancer hallmark. B, The box plots show the absolute values of PCC for GLCRPs in each cancer hallmark of OSCC. C, The point plot shows the GLCRPs in cancer hallmark genome instability and mutation. The *x*‐axis and *y*‐axis represent *P*‐values of the Fisher's test and PCC values of genes and lncRNAs

GLCRPs were identified based on the co‐expression and co‐occurrence of mutations (see Section 2). We identified some GLCRPs for ten cancer hallmarks in OSCC (Figure 2A). The cancer hallmark insensitivity to antigrowth signals had the most GLCRPs (1295). Fewer genes cooperated with more lncRNAs to play their roles in cancer hallmarks. For example, 99 OSCC‐specific genes cooperated with 544 lncRNAs to form 1236 GLCRPs in the cancer hallmark self‐sufficiency in growth signals. The correlations between genes and lncRNAs in GLCRPs for each cancer hallmark were strong (Figure 2B, Table S3). The average PCC absolute value for GLCRPs in most cancer hallmarks was approximately 0.4. Moreover, the genes and lncRNAs in GLCRPs also showed strong co‐occurrence of mutations. For example, in the cancer hallmark genome instability and mutation, the gene SMC1B and lncRNA MIR9‐3HG showed both strong co‐expression (PCC = 0.76) and co‐occurrence (*P*‐value = .008) of mutations in OSCC. The results indicate that the partnerships of genes and lncRNAs in GLCRPs are seen at the mutation and expression levels.

### 3.3 The common and specific features of GLCRPs for cancer hallmarks in OSCC

**FIGURE 3:**
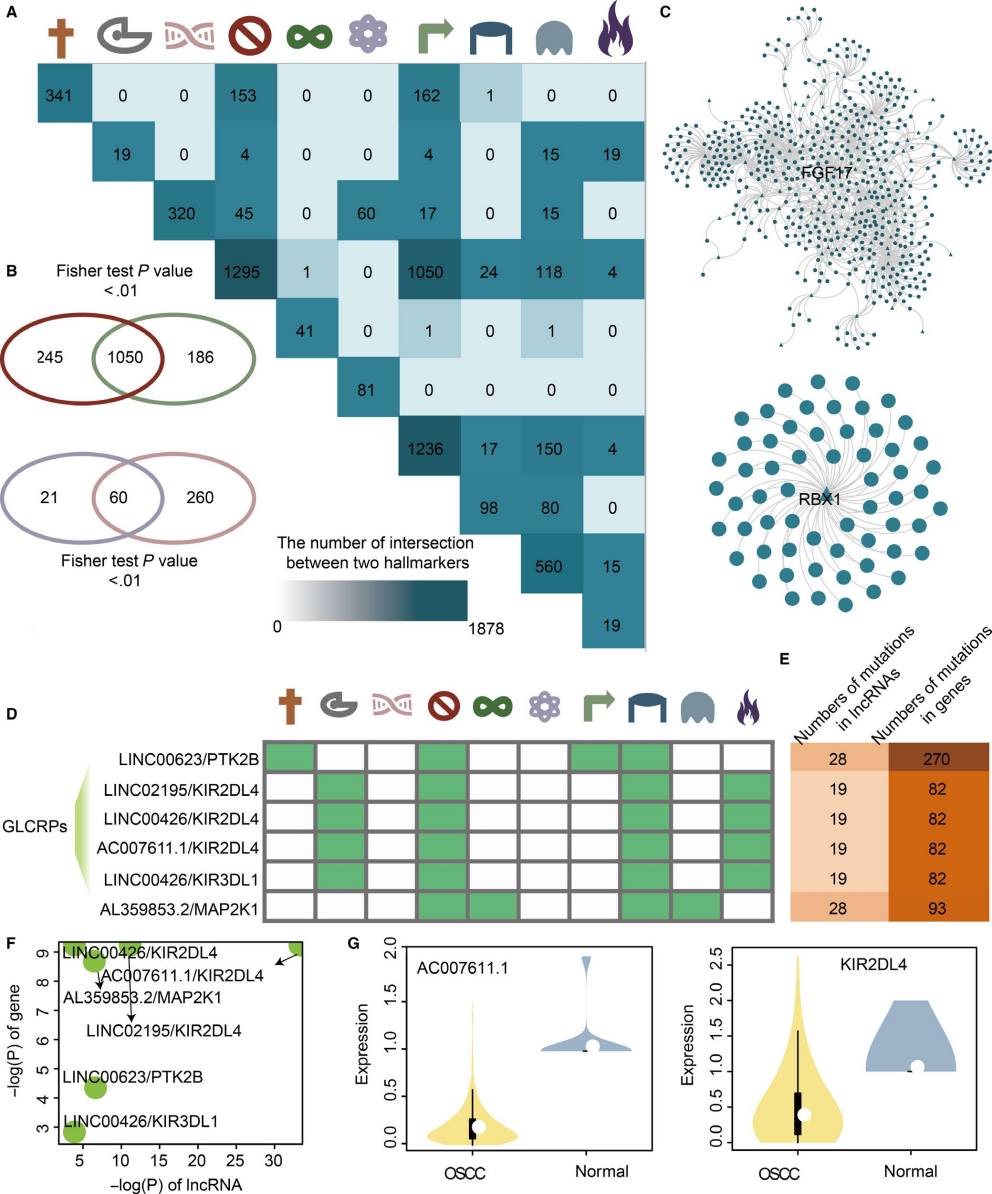
Common and specific features for any two cancer hallmarks based on GLCRPs in OSCC. A, The chart shows the common GLCRPs between any two cancer hallmarks. The darker colour represents more common GLCRPs. B, The Venn diagrams show the intersection of two cancer hallmarks. C, The two networks show the common GLCRPs in OSCC. D, The GLCRPs for OSCC that appeared in multiple cancer hallmarks. E, The darker colour represents more mutations in lncRNAs and genes in OSCC. F, The point plot shows some GLCRPs with corresponding *P*‐values of differentially expressed genes and lncRNAs. G, The violin plots show the expression of AC007611.1 and KIR2DL4

The common GLCRPs between any two kinds of cancer hallmarks are used to characterize the common and distinct features of cancer hallmarks in OSCC. We discover that some cancer hallmarks share a large number of GLCRPs; however, the opposite is the case in some other cancer hallmarks (Figure 3A). For example, the cancer hallmarks self‐sufficiency in growth signals and insensitivity to antigrowth signals significantly (*P*‐value < .01) shared common GLCRPs in OSCC (Figure 3B, Table S4). Cancer hallmark genome instability and mutation and reprogramming energy metabolism also significantly (*P*‐value < .01) shared common GLCRPs in OSCC (Table S4). The core genes of these two common network are FGF17 and RBX1. However, there were no common GLCRPs between the cancer hallmark reprogramming energy metabolism and any other cancer hallmarks in OSCC. Two common GLCRP networks between self‐sufficiency in growth signals and insensitivity to antigrowth signals and reprogramming energy metabolism and genome instability and mutation were constructed (Figure 3C). Some GLCRPs that participate in multiple cancer hallmarks were discovered (Figure 3D). For example, the GLCRP including the lncRNA LINC00623 and the gene PTK2B participated in four cancer hallmarks, including evading apoptosis, insensitivity to antigrowth signals, self‐sufficiency in growth signals and sustained angiogenesis. The lncRNAs and genes in these GLCRPs that participated in multiple cancer hallmarks were all mutated in many OSCC samples (Figure 3E). These genes and lncRNAs were significantly differentially expressed in OSCC samples (Figure 3F). For example, the lncRNA AC007611.1 and the gene KIR2DL4 were all down‐regulated in OSCC samples (Figure 3G). All the results indicated that cancer hallmarks show common and specific features of GLCRPs in OSCC. The corresponding relations of lncRNA name and Ensembl ID were shown in Table S5.

### 3.4 Cancer hallmark‐associated GLCRP networks show complex patterns and specific functions in OSCC

**FIGURE 4:**
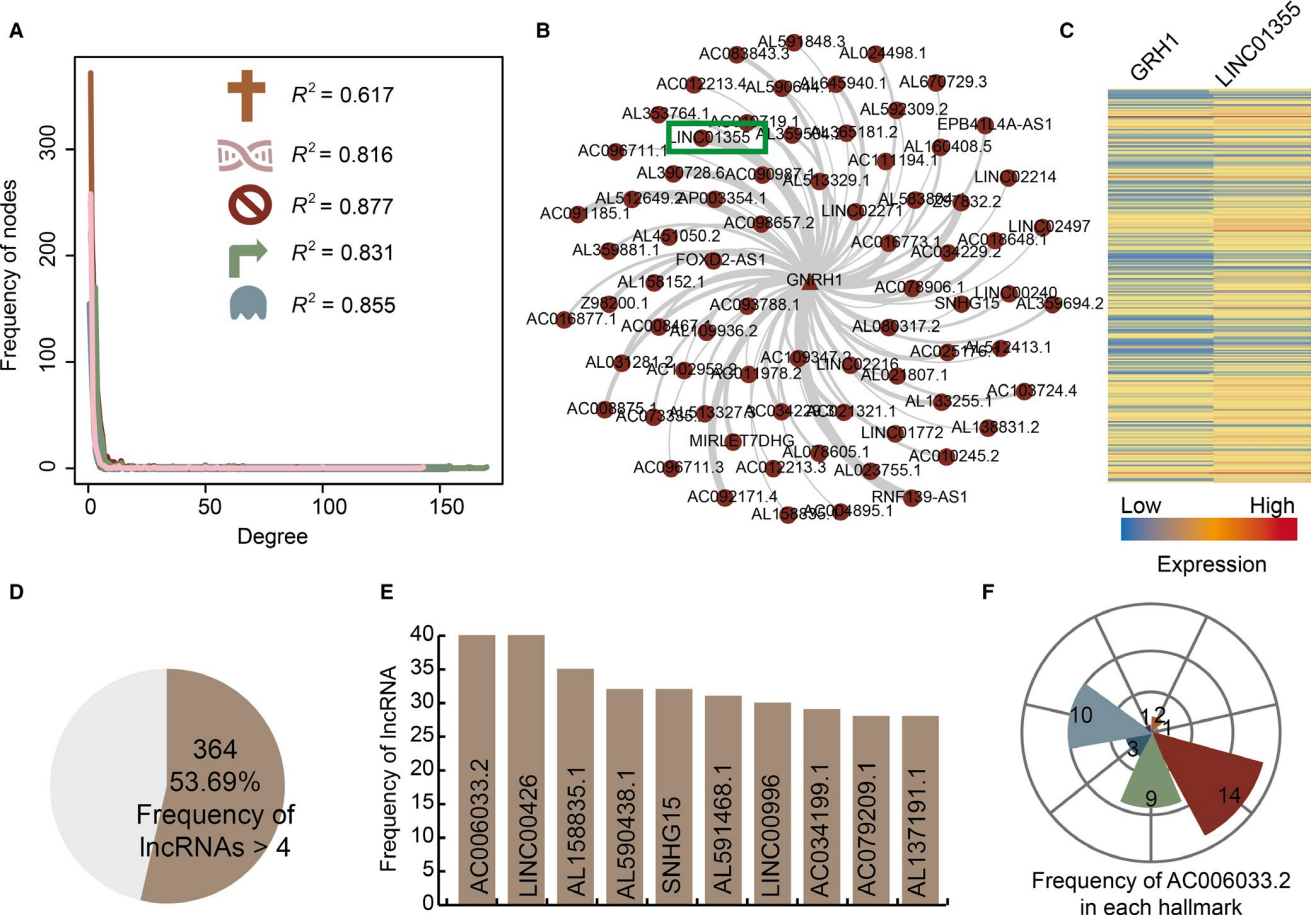
GLCRP networks for each cancer hallmark in OSCC. A, The plots show the degree distribution of GLCRP networks in each cancer hallmark. B, A GNRH1‐associated sub‐network extracted from the insensitivity to antigrowth signals GLCRP network. C, The heat map shows expression of the gene GRH1 and lncRNA LINC01355. D, The pie chart shows the percentages of lncRNAs with frequencies >4. E, The top ten lncRNAs with the highest frequency. The *y*‐axis represents the frequency of lncRNAs. F, The rose diagram shows the frequency of the lncRNA AC006033.2 in each cancer hallmark

To further depict the features of GLCRPs for each cancer hallmark in OSCC, we constructed five cancer hallmark‐associated GLCRP networks. All the networks showed that fewer genes cooperated with multiple lncRNAs in OSCC (Figure S1). All the degree distributions showed a scale‐free network structure and indicated that these networks were significant biological networks (Figure 4A). The *R* ^2^ values of five associated cancer hallmarks were .616, .731, .763, .694 and .738. The cancer hallmark insensitivity to antigrowth signals GLCRP network had the most nodes and edges. The gene GNRH1 had the highest degree (74), and a GNRH1 related sub‐network was extracted from the global cancer hallmark insensitivity to antigrowth signals GLCRP network (Figure 4B). The gene GNRH1 and lncRNA LINC01355 showed strong co‐expression (PCC = 0.75, Figure 4C). For all the cancer hallmark‐associated GLCRP networks, 53.69% of lncRNAs cooperated with more than four genes in OSCC (Figure 4D). The top ten lncRNAs, including AL133215.2, LINC01534, AC092171.4, LINC02560, SNHG15 and so on, with the highest frequency in all the networks are shown (Figure 4E). The lncRNAs AC006033.2, LINC00426, AL158835.1, AL590438.1, SNHG15, AL591468.1, LINC00996, AC034199.1, AC079209.1 and AL137191.1 cooperated with 2, 1, 14, 9, 3, 10 and 1 genes in the cancer hallmarks evading apoptosis, evading immune detection, insensitivity to antigrowth signals, self‐sufficiency in growth signals, sustained angiogenesis, tissue invasion and metastasis and tumour‐promoting inflammation. This result indicates that some lncRNAs play essential roles in cancer hallmark‐associated GLCRP networks for OSCC.

### 3.5 Prognostic benefits of cancer hallmark‐associated GLCRPs in OSCC

**FIGURE 5:**
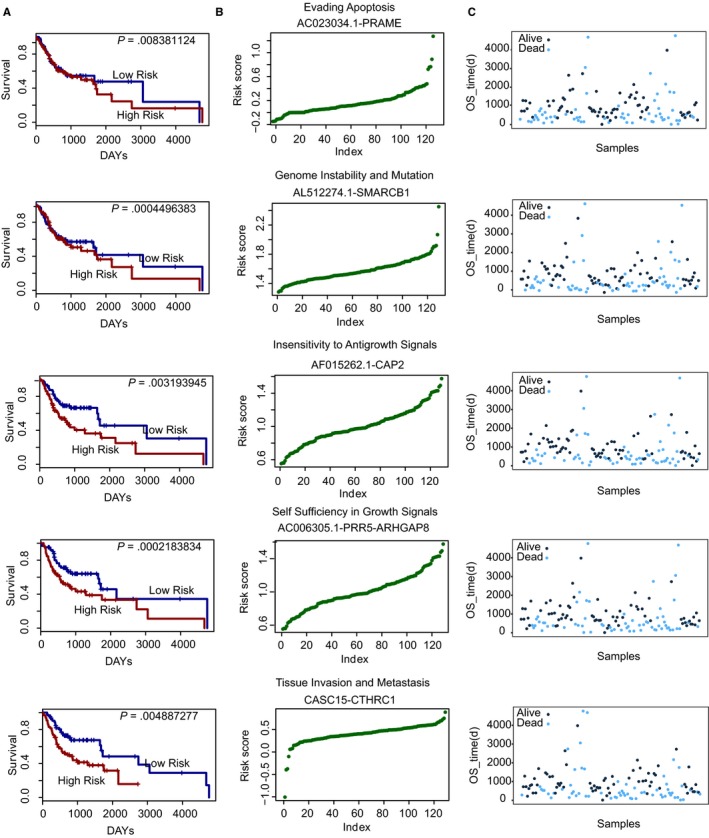
Prognostic analysis of cancer hallmark‐associated GLCRPs in OSCC. A, The Kaplan‐Meier curve for the overall survival of two OSCC groups with high‐ and low‐risk scores. The difference between the two curves was evaluated by a two‐sided log‐rank test. B, The risk‐score distribution of the genes and lncRNAs in each cancer hallmark‐associated GLCRP. C, The patient survival status of the genes and lncRNAs in each cancer hallmark‐associated GLCRP

To evaluate the potential value of GLCRPs as prognostic biomarkers in OSCC, we developed a risk‐score formula according to the expression of the gene and lncRNA expression in each GLCRP to generate OS (overall survival) prediction (see Section 2). The median value of risk scores is considered a cut‐off point to test the survival of OSCC patients. The OSCC patients were divided into high‐ and low‐risk groups based on the above risk scores. In total, 365 (9.1% of all GLCRPs in all hallmarks) GLCRPs were significantly associated with survival in OSCC patients. In five cancer hallmarks, some GLCRPs were significantly related with survival and could be used as candidate prognostic biomarkers (Figure 5A). Moreover, OSCC patients in the high‐risk group had significantly shorter median OS than those in the low‐risk group. The OSCC patients were grouped based on the risk scores of these GLCRPs (Figure 5C,D). The results show that some GLCRPs could collectively influence OSCC patient survival and maybe act as specific prognostic biomarkers.

## 4 DISCUSSION

The present study provides novel insights into the mechanism and treatment of OSCC by exploring the functional significance and molecular mechanism of GLCRPs associated with cancer hallmarks following expression pattern, somatic mutation and CNV data. Accumulating evidence has shown that many lncRNAs contribute to cancer hallmarks.^27^, ^28^ The cancer hallmark‐based method is assigned to explore the roles of lncRNAs in OSCC, and this kind of method is used to identify driver genes in other cancers.^29^, ^30^ In our analysis, we identified some key GLCRPs for each cancer hallmark in OSCC. In addition, the common and specific features for any two kinds of cancer hallmarks were analysed based on GLCRPs. There are 1050 common GLCRPs between the cancer hallmarks insensitivity to antigrowth signals and self‐sufficiency in growth signals. These two cancer hallmarks are both related to tumour growth. The hallmark‐associated GLCRPs generated in this study could facilitate the experimental exploration of lncRNAs in cancer pathogenesis.

Our results demonstrate that the genes and lncRNAs in GLCRPs show strong association on both the mutation and expression levels. The recent application of massively parallel next‐generation sequencing to a growing number of cancer genomes has revealed abundant mutually co‐occurring genetic alteration events.^31^, ^32^, ^33^ Co‐occurrence of mutations indicates that two genes (or other molecules) maybe cooperate to play their roles. Co‐occurrence of mutations is a common and informative phenomenon during cancerogenesis, which helps to uncover novel drivers, decipher their downstream functions and even provide novel treatment methods in cancer. For example, PC9 lung adenocarcinoma cells harbour an activating EGFR exon 19 in‐frame deletion and are naturally sensitive to the EGFR inhibitor erlotinib. Variants such as mutant KRAS that re‐activate downstream signalling pathways can rescue the erlotinib‐induced lethality in this cell type.^34^ In addition, previous study also used the method which lnRNAs were mutually exclusive with well‐known cancer driver genes to distinguish driver lncRNA from passengers.^35^ In the present study, the co‐occurrence of mutations was used to explore the cooperative mechanism of genes and lncRNAs in OSCC. This provides us with important implications in the functional exploration of lncRNAs in cancer pathogenesis. Survival analyses were also performed for GLCRPs associated with cancer hallmarks in OSCC based on an integrative approach. The integration of two genes for analysing survival has been applied in studies about ceRNAs in cancer.^36^ We improved the integrated survival analysis method by randomly dividing the OSCC patients into two independent groups. One group was used for training coefficients of genes and lncRNAs associated with prognosis. The other group was used to obtain risk scores based on the above coefficients and to perform survival analysis. This improved approach avoided the overfitting phenomenon. There are 365 prognosis‐associated GLCRPs that were identified in OSCC and could be candidate prognostic biomarkers for OSCC. In addition, we performed the process of survival analysis for 100 times and these 365 prognosis‐associated GLCRPs were still significant at least 70 times. Although TCGA data set included large sample size, accurate and comprehensive data, we also validated the results in an independent data set from GEO. There were 284 genes and lncRNAs in GLCRPs could be found in this data set and 201 (more than 70%) of them were significantly differential expressed. The results indicated that our analysis was reliable. The data of TCGA are updated constantly, and future work could focus on further validating GLCRPs using updated TCGA data sets or more samples. In addition, more computational methods about differential and co‐expressed analysis for gene and lncRNA should be added to improve the results.

Overall, some OSCC‐specific cancer hallmark‐associated genes and lncRNAs were identified. Based on these OSCC‐specific cancer hallmark‐associated genes and lncRNAs, some GLCRPs were discovered following the co‐expression and co‐occurrence of mutations. Common and specific features for any two cancer hallmarks were depicted in OSCC based on GLCRPs. GLCRP networks for each cancer hallmark were constructed and analysed in OSCC. Survival analyses indicated that GLCRPs could be potential prognostic biomarkers for OSCC. In summary, our study leads to a novel starting point for future functional explorations, the identification of biomarkers and lncRNA‐based targeted therapy for OSCC.

## CONFLICT OF INTEREST

The authors declare that they have no conflicts of interest.

## AUTHOR CONTRIBUTIONS

HYW and YDY conceived and designed the experiments; LK, SLJ, MJ and ZTS analysed the data; and LK and SLJ wrote the manuscript.

## Supporting information

Figure S1

Table S1

Table S2

Table S3

Table S4

Table S5

## Data Availability

The data that support the findings of this study are available from the corresponding author upon reasonable request.
