# Supplementary figures and images for: Identification of cancer hallmark‐associated gene and lncRNA cooperative regulation pairs and dictate lncRNA roles in oral squamous cell carcinoma
Source: J Cell Mol Med. 2020 Mar 23;24(9):5213–23. doi: 10.1111/jcmm.15174 (PMC7205782; doi:10.1111/jcmm.15174)

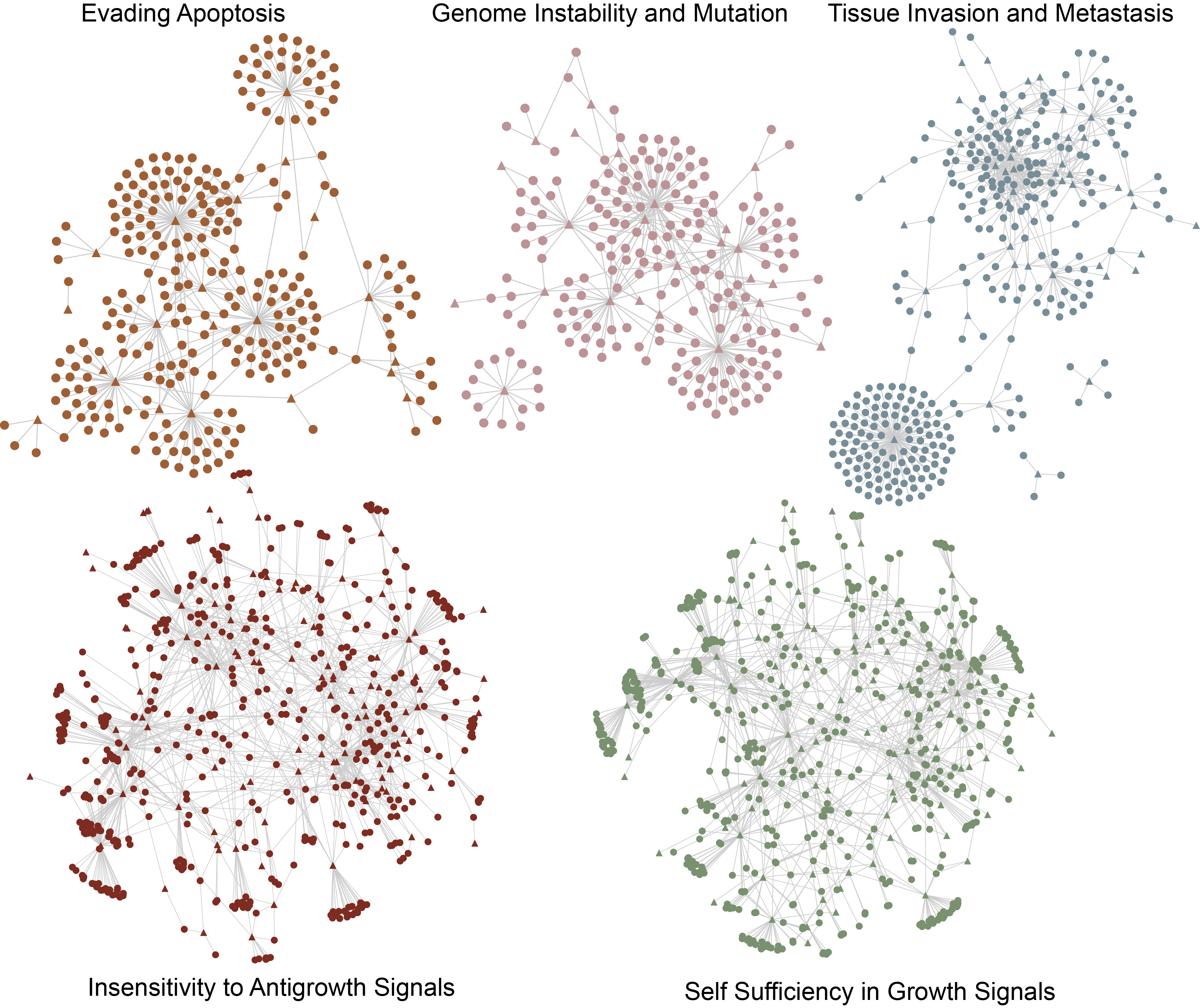

Supplement: Supplementary file 1 — Figure S1 [file JCMM-24-5213-s001.tif]
